# A Case of Bilateral Dens Invaginatus Treated Using the Prophylactic Approach

**DOI:** 10.7759/cureus.66132

**Published:** 2024-08-04

**Authors:** Vaishnavi Patekar, Neha Pankey, Manoj Chandak, Priyanka P Madhu, Monika Khubchandani, Mrunal Meshram, Smruti S Saoji

**Affiliations:** 1 Pediatric and Preventive Dentistry, Sharad Pawar Dental College and Hospital, Datta Meghe Institute of Higher Education and Research, Wardha, IND

**Keywords:** enamel organ, composite restoration, prophylactic management, bilateral lateral incisors, dens invaginatus

## Abstract

Dens invaginatus (DI) is a rare developmental defect in dentistry that results from invagination of the enamel organ into the dental papilla during tooth formation. However, such morphology presents cases that challenge treatment and diagnosis because of the morphology of the canal. The present study reports a case of DI in a 12-year-old boy showing a very unusual clinical and radiographic appearance of maxillary lateral incisors. The flowable composite was used to seal the invagination, and an etchant and a bonding agent were used as part of the preventative or prophylactic clinical therapy that was implemented in this instance. This offers a secure and efficient substitute therapy. This method has the potential to yield the greatest results for patients by combining expertise from endodontics and restorative dentistry.

## Introduction

An uncommon dental disorder called dens in dente, or dens invaginatus (DI), is characterised by the invagination of the enamel organ into the tooth papilla during the soft tissue stage of development. This condition appears in teeth at the morpho-differentiation stage of development [1]. Between 0.3% and 10% of people have DI. Permanent upper lateral incisors exhibit the highest frequency of DI, followed by maxillary central incisors [2]. It is uncertain what causes dens invaginatus. Numerous hypotheses have been issued to determine the cause of DI. Kronfeld proposed that the invagination is caused by a localised failure of the enamel epithelium inside the body, with the normal epithelium around the static region continuing to expand and engulf it [3]. Genetic factors are also believed to be the cause of DI [4]. According to Oehlers, the enamel organ becomes distorted throughout tooth development, and when a segment of the enamel organ protrudes thereafter, an enamel-lined canal emerges, eventually finishing at the cingulum or, on occasion, the incisal tip. The latter might be attached to an irregularly shaped crown [5,6].

The classification of DI given by Oehlers is widely used [5,6]. It includes Type I, where the invagination is very small and walled with enamel; it is contained inside the tooth's crown and does not go past the external amelo-cemental junction. In Type II, the enamel-lined invagination, with no connection with the periodontal ligament, stays inside the root canal and expands into the pulp chamber. In Type IIIA, the invagination, following its progression into the root, forms a pseudo-foramen by interacting laterally with the periodontal ligament area. The pulp is compacted within the root with no interactions with the outside environment. In Type IIIB, the apical foramen is where the invagination meets the periodontal ligament after extending into the root. Typically, there is no interaction with the pulp.

In this report, a case of bilateral DI is presented, with Type I involving the upper right lateral incisor (12) and Type II involving the upper left lateral incisor (22). This condition can lead to pulpal and periapical infections. The management of DI depends upon the pulpal status and anatomy of the tooth. For Oehlers’ Type I and Type II DI with a healthy pulp, the condition was treated by preventive or prophylactic management. According to Shekhar et al., prophylactic treatment can be considered as the preferred mode of approach with a possible Oehlers’ Type I and Type II invagination with the objectives of preserving pulp vitality, preventing caries and meeting esthetic and occlusal requirements [7].

## Case presentation

**Figure 1:**
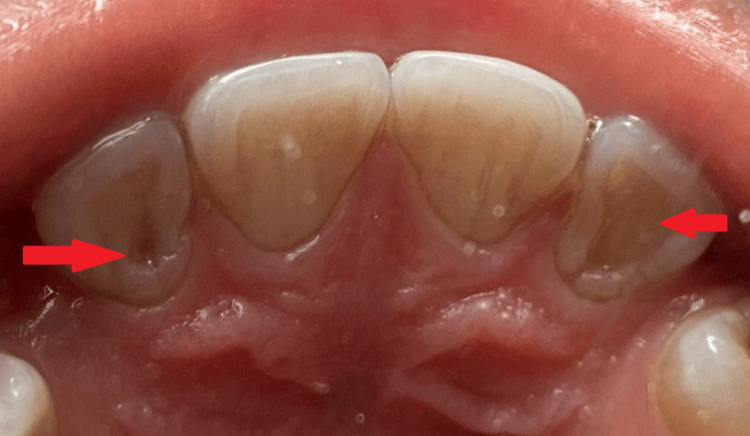
Clinical examination shows dens invaginatus on 12 and 22

A 12-year-old boy presented with the chief complaint of food lodgement in the upper frontal region of the jaw in the past one week. Both dental and medical histories were non-existent. An extraoral examination revealed a normal facial height and facial symmetry, competent lips and a bilaterally synchronous movement of the temporomandibular joint with no deviation in opening and closing movements. On intraoral examination, food lodgement was seen on the palatal surface of the upper right and left lateral incisors. Yellowish-grey discolouration and a deep anatomic pit on the palatal surface of 12 and 22 were seen. There were no cavities and no restorations on the tooth (Figure 1).

**Figure 2:**
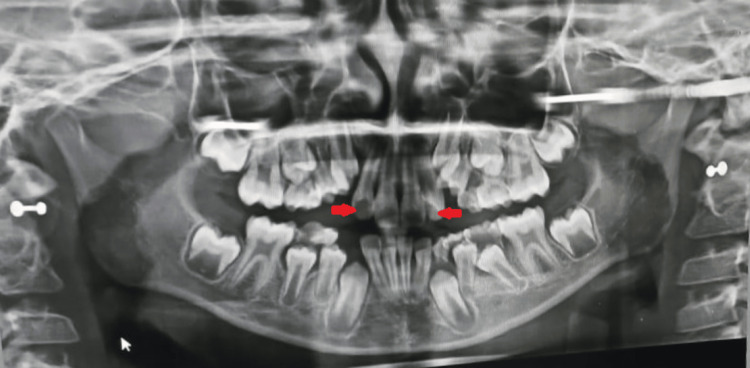
Panoramic radiograph showing upper bilateral dens invaginatus

Teeth showed no sensitivity on horizontal and vertical percussion. The periodontal probing depth was within normal limits. Both overbite and overjet were normal. The dentition showed a Class 1 molar relationship on both sides. A panoramic (orthopantomograph, or OPG) radiograph was taken to acquire a comprehension regarding the morphology and position of teeth in the maxilla. The radiographic evaluation of 12 and 22 showed periapical radiolucency with an ill-defined border and an invagination into the tooth's pulp chamber (Figure 2).

Upon clinical and radiographic examination, the diagnosis was made as Oehlers' Type I DI for 12 and Type II DI for 22. The decision was made to attempt to treat the tooth with a prophylactic or preventive approach using composite restoration application on the invagination. The objectives of the treatment are preventive management of dens invaginatus with a preventive approach, a clear operative area, less discomfort to the patient and a good outcome of the procedure.

**Figure 3:**
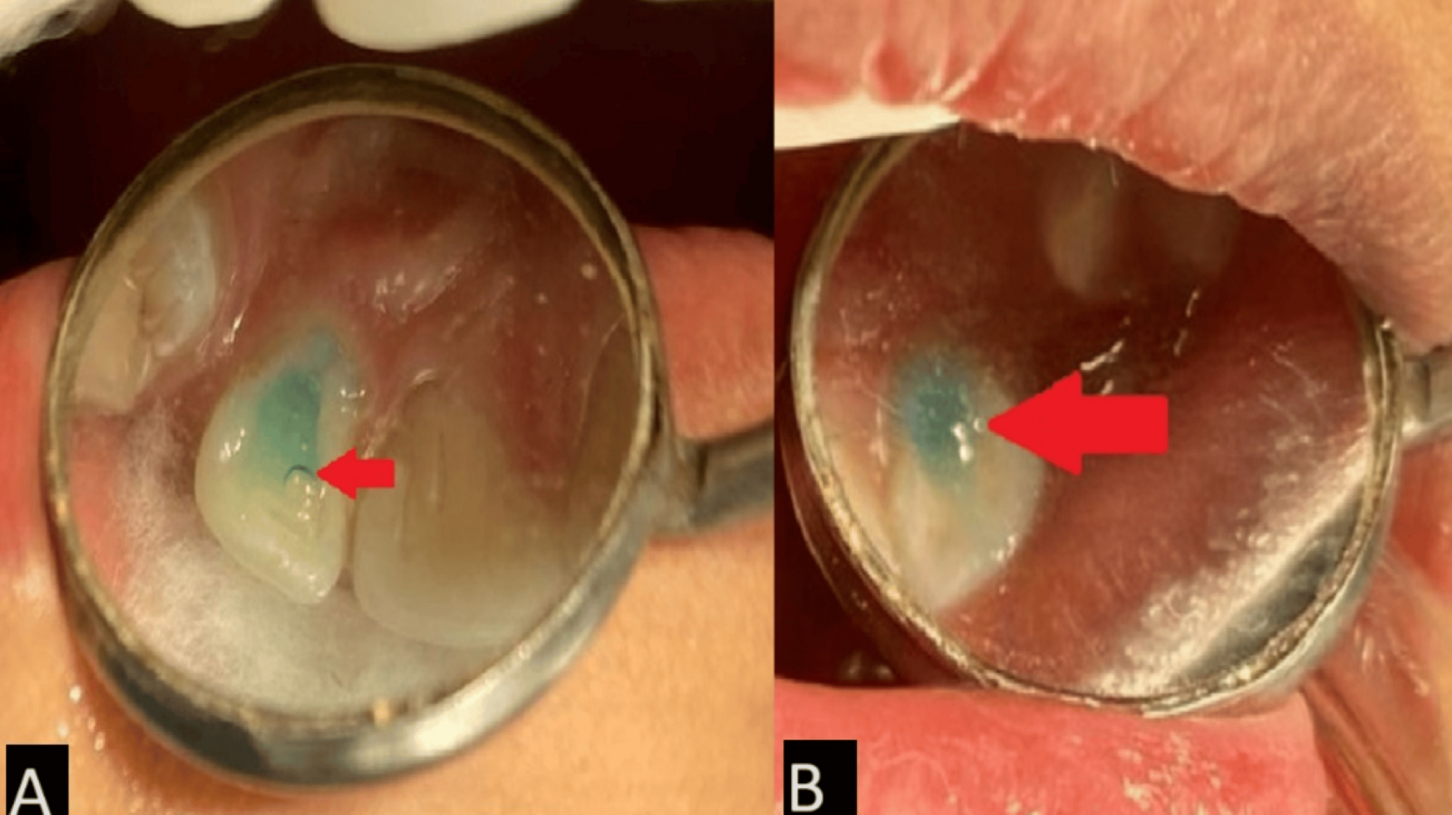
Application of the etching gel over the invagination on (A) 12 and (B) 22

The invagination permits the entrance of irritants into the tooth and makes the site prone to infection. The treatment includes prophylactic sealing of the invagination using composite resin [8]. An informed consent was taken from patient's parents before starting the procedure. Full-mouth ultrasonic scaling (supragingival and subgingival scaling) was carried out to remove plaque and calculus using an ultrasonic scaler (Woodpecker Ultrasonic Scaler UDS-J; Guilin Woodpecker Medical Instrument Co., Ltd., Guilin, China). Etching was done using 37% orthophosphoric acid (Tetric-N-Etch; Ivoclar Vivadent, Zurich, Switzerland) for 10-15 seconds on 12 and 22 (Figure 3) followed by application of the bonding agent (Tetric-N-Bond; Ivoclar Vivadent). The prophylactic composite restoration (Clearfil AP-X; Kuraray, Co., Ltd., Osaka, Japan) was done on the invagination. The patient reported no pain during treatment.

**Figure 4:**
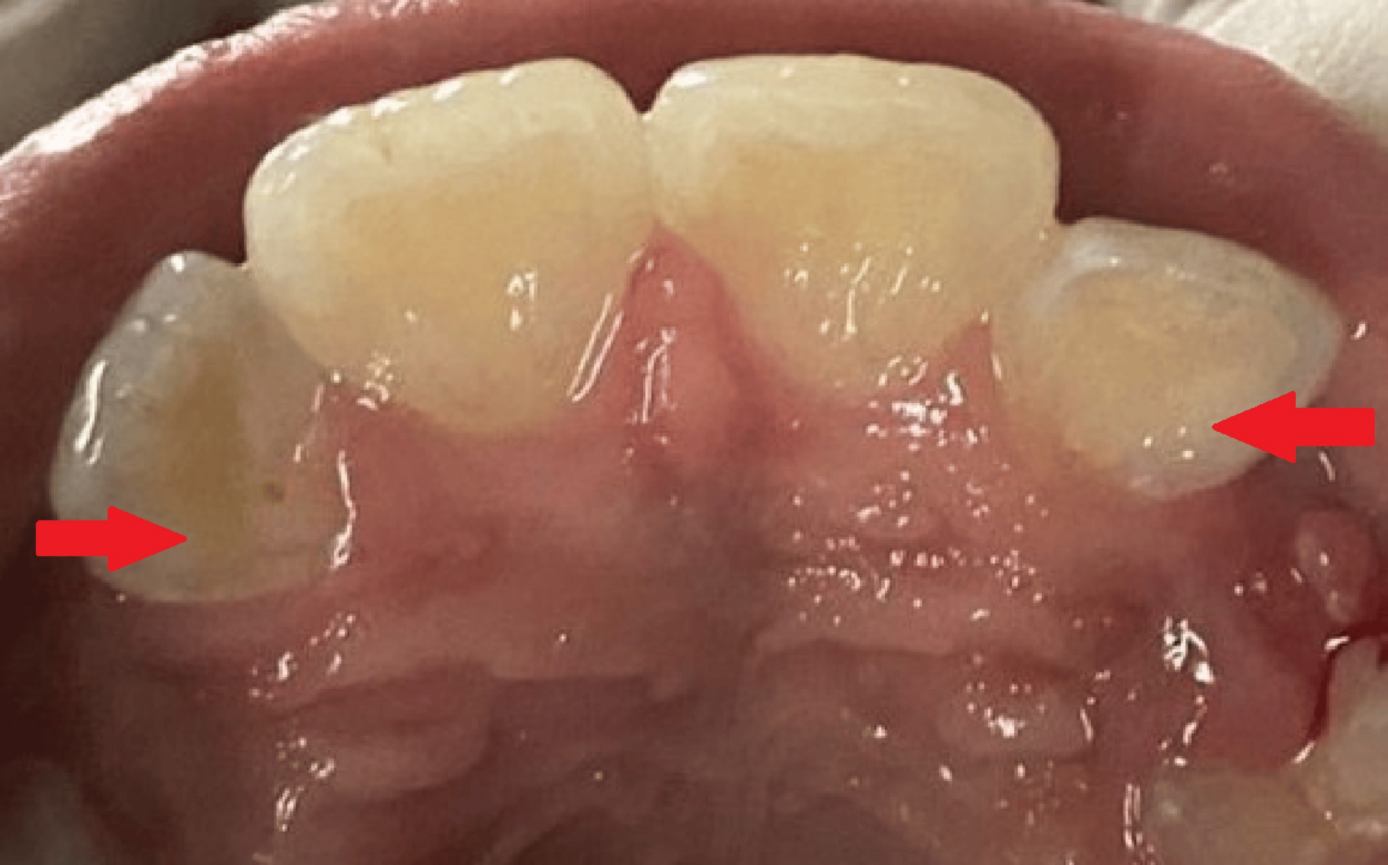
Prophylactic composite restoration used for sealing of the invagination

Following treatment, the patient received all the post-restorative instructions needed to maintain proper dental hygiene, like refraining from eating hard foods for the first day to ensure the filling hardened correctly, brushing and flossing gently around the restored area to maintain oral hygiene, and reaching out to the dentist in the case of any pain or sensitivity; he was subsequently released from the hospital. Additionally, a chlorhexidine mouthwash to be used twice a day for one week was recommended, as discoloration is considered a major concern with its prolonged use. The patient was monitored on a follow-up basis, scheduled every four weeks. The procedure was successful, without any signs of pain or development of discolouration on teeth (Figure 4).

## Discussion

Dens invaginatus is an uncommon dental aberration that develops when the enamel organ infiltrates the dental papilla during the process of tooth formation. The condition results in a deep pit or pocket walled with enamel that permits the entrance of irritants into an area making the site prone to infections like pulpitis and periapical infections. However, in this particular case, DI of the bilateral upper lateral incisors was accidentally identified on periapical x-rays. The pulp was in a good state and there was no abnormality detected on the outer part of the crown, except for the presence of palatal pits in the bilateral upper lateral incisor. Depending on the state of the surrounding tissues and tooth pulp as well as the complexity of the tooth's structure, several approaches are taken to managing DI. For most cases with Type II DI, where pulp necrosis is evident, the most appropriate treatment option is root canal treatment [9]. But, in cases where Oehlers' Type I or II DI is present and the pulp is healthy, it is important to carry out suitable preventive or prophylactic measures [8,10]. In the treatment of dens invaginatus, root canal therapy addresses the internal complexities and prevents infection, making it suitable for severe cases. Conversely, composite restorative treatments focus on repairing external defects and aesthetics, and are generally more appropriate for less severe presentations. In this case, pulp necrosis was absent and the pulp was healthy, so preventive management as the treatment option was chosen. The treatment was also chosen because of the patient's young age and the desire to maintain the tooth's functional and aesthetic state for as long as possible.

Here, clinical management was started using an ultrasonic scaler to remove plaque and calculus thoroughly, particularly in the invaginated areas, thus reducing the risk of bacterial colonization and subsequent caries. The use of an ultrasonic scaler emphasizes its effectiveness in reaching and cleaning complex tooth structures. This helps to keep the working area clean and lowers the chance of infection. Following scaling, a conventional etching gel was applied for 10-15 seconds over the enamel and dentin surfaces followed by the application of bonding agent. This permits the use of less invasive restorative techniques and helps in preserving the natural tooth structure. The invagination was finally sealed with the light-cured composite resin [11,12]. The composite fills in the gaps in the aesthetic and functional appearance of the tooth and it also prevents further complications. This produces beautiful, efficient dental aesthetic results. Routine follow-ups were done to monitor the health of the tooth as a whole and the standard of restoration.

This case reinforces the use of a team approach, where identification of the problem at an early stage, prevention, and follow-up are significant. A positive outcome in this case reminds us of the importance of individualized treatment planning in every patient's unique DI situation. In the future, research and clinical work ought to focus on the development of methods for the optimum diagnosis and management of such cases for enhancing outcomes. This case reiterates the need for highly individualized treatment plans that consider the dens invaginatus anatomy and clinical presentation of each patient. Future research and clinical work should focus on establishing the most effective methods to diagnose and treat such cases to achieve the best possible patient outcomes.

## Conclusions

Dens invaginatus represents one of the more demanding pathologies in dentistry because of its potential clinical manifestation and varied possible complications that may arise. Early diagnosis and appropriate therapy are crucial to avoid serious consequences. In terms of treatment, composite restorations are considered popular because they have several aesthetic rehabilitative advantages. This case demonstrates that teeth with dens invaginatus can be treated with prophylactic or preventive therapy.
